# Analysis of Structural Changes of pH–Thermo-Responsive Nanoparticles in Polymeric Hydrogels

**DOI:** 10.3390/gels10080541

**Published:** 2024-08-20

**Authors:** Lazaro Ruiz-Virgen, Miguel Angel Hernandez-Martinez, Gabriela Martínez-Mejía, Rubén Caro-Briones, Enrique Herbert-Pucheta, José Manuel del Río, Mónica Corea

**Affiliations:** 1 Laboratorio de Investigación en Polímero y Nanomateriales, ESIQIE, Instituto Politécnico Nacional, Av. Luis Enrique Erro S/N, Unidad Profesional Adolfo López Mateos, Zacatenco, Alcaldía Gustavo A. Madero, Mexico City 07738, Mexico; lazaro1990@hotmail.com (L.R.-V.); mamtz6046@gmail.com (M.A.H.-M.);; 2 Escuela Superior de Ingeniería Mecánica y Eléctrica, ESIME, Instituto Politécnico Nacional, Av. Luis Enrique Erro S/N, Unidad Profesional Adolfo López Mateos, Zacatenco, Alcaldía Gustavo A. Madero, Mexico City 07738, Mexico; 3 Departamento de Química Orgánica, Escuela Nacional de Ciencias Biológicas, ENCB, Instituto Politécnico Nacional, Prolongación de Carpio y Plan de Ayala S/N, Colonia Santo Tomás, Mexico City 11340, Mexico; 4 Departamento de Ingeniería en Metalurgia y Materiales, ESIQIE, Instituto Politécnico Nacional, Av. Luis Enrique Erro S/N, Unidad Profesional Adolfo López Mateos, Zacatenco, Alcaldía Gustavo A. Madero, Mexico City 07738, Mexico; jm.delrio.garcia@gmail.com

**Keywords:** stimuli-responsive hydrogels, polymeric nanoparticles, drug delivery systems, (LCST) lower and (UCST) upper critical solution temperature, polyelectrolytes and particle diffusion coefficient (*D*)

## Abstract

The pH- and thermo-responsive behavior of polymeric hydrogels $\left(M_{C}-co-M_{A}\right)$ have been studied in detail using dynamic light scattering $\left(DLS\right)$, scanning electron microscopy$\;\left(SEM\right)$, nuclear magnetic resonance (^1^H NMR) and rheology to evaluate the conformational changes, swelling–shrinkage, stability, the ability to flow and the diffusion process of nanoparticles at several temperatures. Furthermore, polymeric systems functionalized with acrylic acid $\left(M_{C}\right)$ and acrylamide $\left(M_{A}\right)$ were subjected to a titration process with a calcium chloride $\left({CaCl}_{2}\right)$ solution to analyze its effect on the average particle diameter$\;\left(D_{z}\right)$, polymer structure and the intra- and intermolecular interactions in order to provide a responsive polymer network that can be used as a possible nanocarrier for drug delivery with several benefits. The results confirmed that the structural changes in the sensitive hydrogels are highly dependent on the corresponding critical solution temperature $\left(CST\right)$ of the carboxylic (–COOH) and amide (–CONH_2_) functional groups and the influence of calcium ions $\left({Ca}^{2+}\right)$ on the formation or breaking of hydrogen bonds, as well as the decrease in electrostatic repulsions generated between the polymer chains contributing to a particle agglomeration phenomenon. The temperature leads to a re-arrangement of the polymer chains, affecting the viscoelastic properties of the hydrogels. In addition, the diffusion coefficients $\left(D\right)$ of nanoparticles were evaluated, showing a closeness among with the morphology, shape, size and temperature, resulting in slower diffusions for larger particles size and, conversely, the diffusion in the medium increasing as the polymer size is reduced. Therefore, the hydrogels exhibited a remarkable response to pH and temperature variations in the environment. During this research, the functionality and behavior of the polymeric nanoparticles were observed under different analysis conditions, which revealed notable structural changes and further demonstrated the nanoparticles promising high potential for drug delivery applications. Hence, these results have sparked significant interest in various scientific, industrial and technological fields.

## 1. Introduction

In recent years, the synthesis of materials on a nanoscale have been extensively studied for different applications because they have the potential to improve the technology and functionality of several products in many fields, such as aerospace, chemical engineering, construction, environment, biomedicine, among others [1,2].

Additionally, technology and engineering have played an important role in the synthesis and characterization of new advanced materials, known as smart or intelligent materials, which are becoming very popular. These materials can modify their shapes, dimensions and mechanical properties when an external stimulus is applied [2].

In general, smart materials can be divided into the following four groups according to their composition: ceramics, metallic, inorganic and polymeric smart materials [2,3]. Among them, polymer hydrogel systems that contain functional groups in their structures have been used as adhesives, fat replacers, protective structures, drug carriers, textures modifiers and civil infrastructure, also providing strategies for the design of new engineered systems that can reduce or increase porosity [4]. Some hydrogel particles under acid conditions can improve resistance, stiffness, wettability, surface functionalization, emulsion stability or create polymeric biomaterials [4,5]. In addition, these properties depend on their preparation conditions, size distribution, morphologies, composition, structure, high stability, permeation, flexibility, solubility, hydrophilicity, degradability, biocompatibility and responsiveness to specific environments [6,7].

In particular, polymeric hydrogels that are sensitive to physicochemical changes within their surrounding environment have been developed to detect physical (light, electricity, deformation, ultrasound, temperature, magnetic fields and mechanical stress), chemical (pH, ionic strength and redox potential) and biological (enzymes, antigens and ligands) stimuli, and they can react with self-assembly or changes in their properties [8].

Specifically, pH responsive polymeric hydrogels are materials that include in weak acid or basic groups in their structures along the polymer backbone [8]. When the pH of the medium is modified, polymer functional groups can accept or donate protons $\left(H^{+}\right)$, changing the ionization degree and the net charge on the polymer chains [8]. Thereby, this behavior leads to the alteration of their surface activity, solubility, hydrodynamic volume and conformation [8]. On the other hand, temperature responsive polymer hydrogels exhibit a critical solution temperature $\left(CST\right)$ and intra- and intermolecular interactions in aqueous medium [9]. At CST, the polymer solutions undergo a volume phase transition between an extended and compacted coil state which is associated with drastic changes in the solvation of the polymer and the free energy of the polymer–solvent system [10]. Consequently, the following two different thermos-responsive behaviors are reported: the Lower Critical Solution Temperature$\;\left(LCST\right)\;$and the upper critical solution temperature $\left(UCST\right)$ [11]. The LCST is usually referred as an entropic process where the formed bonds between the polymer chains and the solvent molecules are balanced by the order in the solvent molecules and the decrease in the mixing entropy [11]. Therefore, below the LCST, the polymer chains stretch out and swell. Moreover, in increasing the temperature of the system, the entropy becomes predominant, leading to a positive free energy of mixing. That is, above the LCST the chains are hydrophobic and collapse into a condensed conformation [11,12]. The polymer–polymer interactions are thermodynamically more stable than polymer–solvent interactions, contributing to release of the solvent molecules in the bulk and the formation of two phases [11].

In contrast, the UCST is driven by the mixing enthalpy [11]. The cohesive polymer–polymer interactions responsible for the self-association are destabilized above the CST, leading to the formation of one single phase. These interactions can arise from the hydrogen bonding between the polymer side groups or from coulombic forces between polymeric chains combining cationic, anionic or zwitterion interactions [11].

Recently, polymeric hydrogels, having both pH and temperature responsiveness (dual responsive polymers), have been extensively studied and reported in the literature [13]. For instance, thermo-responsive and pH-sensitive hydrogels have been used for in vivo applications, cardiac therapies, intra-cellular drug delivery, bioglue, antibacterial adhesive, biodegradable stents, human tissues, soft robotics, membrane separations, fiber mats, strain sensors, textiles and the oil industry Ref. [14] because they have the possibility of inducing a modification in the polymeric hydration with a temperature gradient and the ability to dissociate into highly charged polymeric molecules, if they are immersed in water or other ionizing solvents [15,16].

In particular, for drug delivery systems of active species into biological applications from pH- and thermo-sensitive polymer hydrogels, certain characteristics are considered, as follows: the ability to achieve a specific and controlled drug release in response to local environmental variations, utilizing physicochemical interactions with the surrounding molecules to be able to link the polymers with a drug through the ionizable groups located along their chemical structure [7,8]. Therefore, in response to the pH and temperature changes, the hydrogen bonds formed between the polymer chains of the hydrogels and the solvent molecules are able to break up, inducing the release of the bioactive compounds as a result of the swelling or shrinking behavior of the material [7,8]. Furthermore, polymeric structures are widely used as delivery vehicles for drugs and various agents due to their size, porosity, permeation, flexibility, hydrophilicity, water content, chemical affinity, dissociation, toxicity, degradation, biocompatibility, adhesion to surfaces, activation energy, adsorption, etc. [7,8,13,14,15,16].

Currently, the characterization and measures of physical and chemical properties of pH- and temperature-sensitive nanoemulsions (10 to 1000 nm) by several techniques contribute to establishing the design and fabrication of nanoparticles by means of novel methods and advanced technologies which are attractive but challenging in regard to scientific and technological interest [17,18]. For this, it is relevant to study the concentration of the solutions, interfacial energies, Brownian motion and the stability of the polymers [17]. For instance, the analysis of the molecular weight, composition structure, dynamics and diffusion properties, such as self-diffusion coefficient $\left(D\right),$ used to determine the size distribution of the polymer particles by nuclear magnetic resonance $\left(NMR\right)$ provides unique information regarding the nature and interactions of the nanopolymers at atomic levels [19,20]. Furthermore, the analysis of the rheological behavior of emulsions throughout parameters such as the purity of chemical components, molecular weight, structure, degradation of the polymer, nanoparticle concentration, solution pH and storage temperature provides critical information for the design, modeling, and processing conditions and synthesis of the materials [18,21,22]. For this reason, the study of the design, synthesis methods, physical–chemical properties and the behavior of polymeric particles holds immense significance due to their wide range of applications, especially in biological targets such as imaging agents, smart sensors, 3D bioprinting, tissue engineering, cancer treatments, transdermal, gene, cells and drug delivery, and even as supracolloidals systems with varying shapes and lengths for building DNA-functionalized molecular blocks, among others [8,13,14,23]. Understanding these aspects is crucial for utilizing these materials efficiently and effectively.

The aim of this research is the synthesis, characterization and analysis of the structural changes in polymeric nanoparticles of hydrogels functionalized with acrylic acid$\;\left(M_{C}\right)\;$and acrylamide$\;\left(M_{A}\right)\;$with two different morphological arrangements. The hydrogels were subjected to specific variations of the pH and temperature of the surrounding environment in order to evaluate their physicochemical response to certain stimuli. The change in the average diameter, stability, tendency to flow and diffusion of the polymeric particles in the medium are a consequence of the processes of hydration, contraction, ionization and the intermolecular interactions between the pH-sensitive (carboxylic, –COOH) and thermo-sensitive (amide, –CONH_2_) functional groups and the solvent molecules induced by the changes in the pH and temperature of the system. The obtained results demonstrated that the polymeric hydrogels$\;\left(M_{C}-co-M_{A}\right)\;$are optimal candidates for the release of drugs in various biomedical therapies for the treatment of several diseases.

## 2. Results and Discussion

### 2.1. Effect of an Electrolyte on the Average Particle Diameter $\left(D_{z}\right)$ and Zeta Potential (ζ) of Poly$\left(M_{C}-co-M_{A}\right)$ Nanoparticles

**Figure 1:**
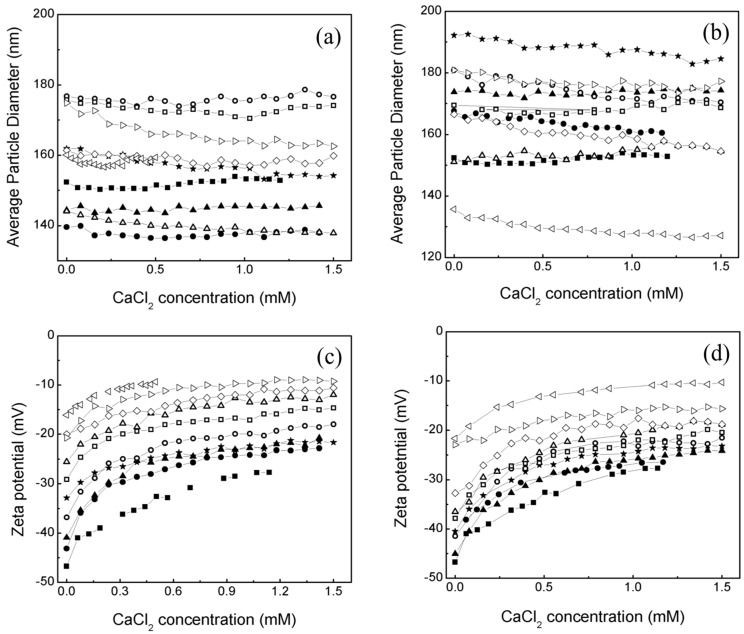

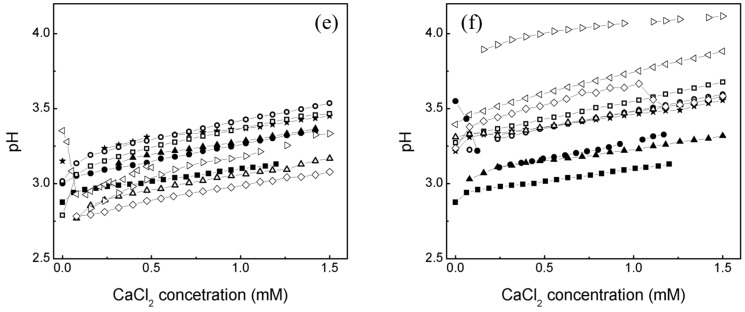
Average particle diameter $\left(D_{z}\right)$, zeta potential (ζ) and pH as function of calcium chloride $\left({CaCl}_{2}\right)$ for: (**a**,**c**,**e**) core−shell and (**b**,**d**,**f**) core with a concentration gradient polymeric particles with the concentrations of (■) 100:0; (●) 90:10; (▲) 80:20; (★) 70:30; (◯) 60:40; (☐) 50:50; (△) 40:60; (◊) 30:70; (▷) 20:80 and (◁) 10:90 of $M_{C}-M_{A}$ (wt.%:wt.%), respectively.

The distribution particle diameter and the zeta potential (ζ) data of all latex measured at temperatures 25 ≤ T/°C ≤ 60 have been discussed in detail elsewhere [22]. Subsequently, to evaluate the behavior of polymeric hydrogels when interacting with an electrolyte, both morphologies of polymeric particles were titrated with a$\;{CaCl}_{2}\;$solution. For that, the measures of average particle diameter $\left(D_{z}\right)$, zeta potential (ζ), and pH were made during all the titration process and the results are compared in Figure 1. The measures were determined at 25 °C and made in triplicate.

It is observed for both morphologies that a particle size of Serie 2 $\left(S_{2}\right)\;$is bigger than a particle size of Serie 1 $\left(S_{1}\right)$; however, in both cases the average particle size does not change much during the titration process (Figure 1a,b). This is attributed to the arrangement of pH (carboxylic, –COOH) and thermo (amide, –CONH_2_) sensitive groups inside the hydrogels.

For the shell of the particles with core–shell morphology, the thermo-sensitive behavior of acrylamide-based polymers exhibits Lower Critical Solution Temperature$\;\left(LCST\right)\;$characteristics that are typical of amide derivatives. As the temperature increases, hydrogen bonding between amide groups is predominant, yet interactions with water molecules persist. This leads to a re-arrangement of hydrogen bonds between the thermo-sensitive groups and solvent molecules. Notably, this phenomenon does not influence the particle size, morphology, or shell thickness but significantly impacts the wettability of the polymer shell [24].

For the core of the particle, a copolymer with pH-sensitive groups was synthesized which, according to the bibliography, exhibits an upper critical solution temperature $\left(UCST\right)\;$close to ~25 °C [25,26]. Polymers that show an upper critical solution temperature$\;\left(UCST\right)\;$in aqueous medium swell in response to increments in the temperature; therefore, dipolar interactions are broken to yield completely solvated and isolated polymer chains and the polymeric hydrogel is mostly hydrophilic above the UCST. Otherwise, as the temperature is reduced, the hydrophobic interactions, which are polymer–polymer and solvent–solvent, are stronger than hydrophilic interactions (polymer–solvent) [11,15,22]. This behavior leads to self-association of the polymer chains, a reduction in polymer size and, thus, to the production of a collapsed non-associated state. This polymer thermo-mechanism is influenced by electrostatics interactions, ionic strength, hydrolysis, polymer concentration and/or the pH of the medium [11,15,22,26].

Copolymers based on acrylic acid $\left(M_{C}\right)\;$and acrylamide$\;\left(M_{A}\right)\;$that exhibit a UCST-like behavior form a polycomplex structure. These bindings provide a non-interrupted linear sequence of bonds that appear between the acrylic acid and the acrylamide (a proton acceptor polymer). These closed cooperative interactions between the molecules, result from hydrogen bonds and can be broken upon heating in aqueous media [22,26]. For this to happen, the pH of the solution also becomes a critical parameter since the interaction occurs below a pH value where the degree of ionization is low enough [22,26]. Hence, the carboxylic group (–COOH) contained in the acrylic acid$\;\left(M_{C}\right)$ is in a non-ionized state. This means that non-covalent interactions between polymer chains remain and, therefore, the particles shrink and the average diameter decreases or remains constant. This is more evident when the concentration of pH-sensitive groups in the particle increases [22,26].

This behavior is also presented for these hydrogels when the pH is lower than four because the ionization degree in a polymer bearing weakly ionizable groups is changed at a specific pH known as pKa. When the pH of the medium is modified, functional groups are capable of accepting or donating protons$\;\left(H^{+}\right)$, changing the degree of ionization and the net charge on the polymer chain [8]. According to Salime Bazban-Shotorbani and their co-workers [27], pH-sensitive groups have a pKa value close to 4.5 [27]. If the pH < pKa then the pH-sensitive groups are predominantly uncharged. Interestingly, these can be observed in the results obtained regarding pH measurements for both morphologies (Figure 1e,f) where the values of the pH during all the titration processes are below 4.5. The pH-thermo responsive mechanism has been explained in a previous work [22].

On the other hand, zeta potential (ζ) is the electrokinetic potential in colloidal systems and its magnitude determines the stability of the particles. Values over the range +30 < ζ/mV < −30 indicate high colloidal stability. Thus, lower ζ values will lead to aggregation, coagulation or flocculation due to van der Waals interparticle attraction [28].

Prior to titration, the initial zeta potential (ζ) values of particles in Serie 1 $\left(S_{1}\right)$ and Serie 2 $\left(S_{2}\right)$ increased from −46.75 mV to −16.10 mV and −46.75 mV to −21.70 mV, respectively, as the concentration of thermo-sensitive groups rose (Figure 1c,d). This suggests that the amide groups remained un-ionized, leading to strong polymer–polymer interactions and minimal repulsion charges on the polymeric chains, resulting in particle agglomeration [29].

Consequently, polymers with a higher proportion of thermo-sensitive groups exhibited greater instability compared to hydrogels with a higher pH-sensitive group content. Following titration, changes in zeta potential values (Figure 1c,d) indicate that the carboxylic and amide groups on the polymeric chains interacted with calcium ions $\left({Ca}^{2+}\right)$. The addition of$\;{CaCl}_{2}\;$electrolyte solution increased the zeta potential values, reducing electrostatic repulsions and decreasing system stability.

### 2.2. Morphology of Poly$\left(M_{C}-co-M_{A}\right)$ Nanoparticles

**Figure 2:**
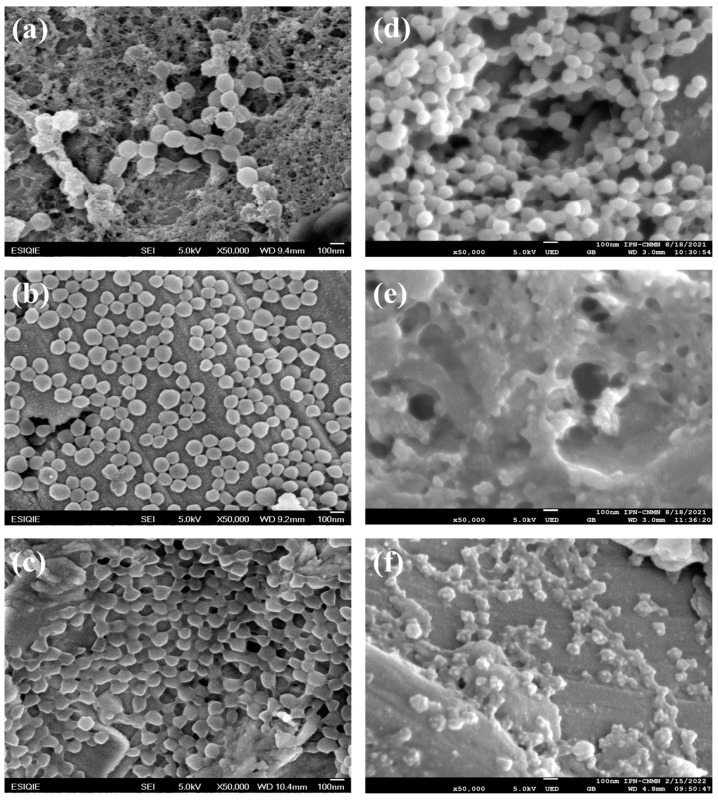
SEM images of polymeric particles poly$\left(M_{C}-co-M_{A}\right)$ of Serie 1: (**a**) 100:0, (**b**) 50:50 and (**c**) 0:100 and Serie 2: (**d**) 90:10, (**e**) 50:50 and (**f**) 0:100 of $M_{C}-M_{A}$ (wt.%:wt.%), respectively.

The morphologies of the polymer nanoparticles poly$\left(M_{C}-co-M_{A}\right)\;$ of Serie 1 $\left(S_{1}\right)$ and Serie 2 $\left(S_{2}\right)$ were also analyzed by SEM (Figure 2). The micrographs showed polymeric hydrogels uniformly copolymerized with pH (carboxylic, –COOH) and thermo (amide, –CONH_2_) responsive groups. In general, the particle surface of poly$\left(M_{C}-co-M_{A}\right)\;$is regular, uniform and very porous. However, if the surface morphologies are compared individually for each concentration, they showed structural changes which depend on the ratio of acrylic acid $\;\left(M_{C}\right)\;$ and acrylamide$\;\left(M_{A}\right)\;$inside the particles and the proposed synthesis process.

According to SEM analysis, nanoparticles with a core–shell morphology were observed with a spherical shape and a very narrow particle size distribution (Figure 2a–c). Specifically, the hydrogels with a high pH-sensitive group concentration showed regular spherical nanoparticles attributed to the high negative surface charge of the ionized carboxyl groups (–COO^–^), (Figure 2a). Also, particles with a 50:50 ratio of $M_{C}-M_{A}$ have a well defined spherical shape, and this corresponds to the equal moiety of carboxylic (–COOH) and amide (–CONH_2_) functional groups pendants in their structure, as is shown in Figure 2b. Otherwise, the polymers with a 0:100 ratio of $M_{C}-M_{A}\;$ have an irregular morphology and disordered structure, and the particles began to interact strongly with each other as the content of the pH-sensitive groups decreases (Figure 2c).

Conversely, SEM images of Figure 2d–f for particles with a gradient concentration indicated closely packed tiny spherical nanoparticles with aggregates in a high proportion of pH- and thermo-sensitive groups $\;{(M}_{C}-co-M_{A})\;$ on the surface. As shown in Figure 2d, the particles with a 90:10 ratio of $\;M_{C}-M_{A}\;$ presented a better size distribution but a similar spherical morphology than those hydrogels with a core–shell structure by the high concentration of pH-sensitive groups on their surface. Nevertheless, the higher ratio of thermo-sensitive groups promoted the chain extension of polymeric segments, generating a mostly porous surface (Figure 2e). This behavior can be explained by the disappearance of smooth planes associated with rising surface protrusions, creases and wrinkles. Furthermore, the polymeric chain length grew enough to fold and collapse on itself, allowing for the formation of inter- and intra-hydrogen bonding between the pH- and thermo-sensitive groups (electrostatic interactions), causing insoluble polymers at ambient temperature and leading to an aggregation of the particles (Figure 2f) [11,30].

This effect could be related to the UCST of the copolymer $\;\left(M_{C}-co-M_{A}\right)\;$ due to the analysis temperature being below upper critical solution temperature (~25 °C); therefore, the polymer depends on hydrogen bonding between polymer side groups for the phase transition and is not completely soluble in water, as is described above [31]. This thermo-sensitivity response of the copolymer is also explained in detail elsewhere [22].

### 2.3. Morphology of Poly$\left(M_{C}-co-M_{A}\right)$ Nanoparticles Titrated with an Electrolyte

**Figure 3:**
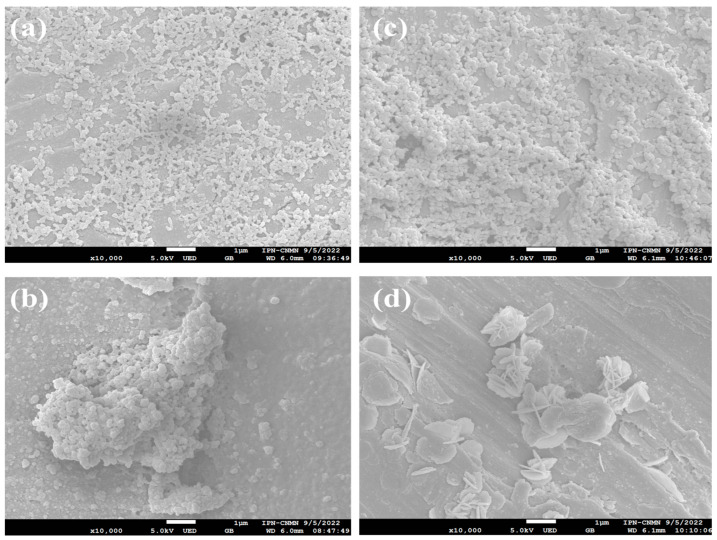
SEM images of polymeric particles poly$\left(M_{C}-co-M_{A}\right)$ with an electrolyte solution added for Serie 1: (**a**) 70:30 and (**b**) 10:90 and Serie 2: (**c**) 70:30 and (**d**) 10:90 of $M_{C}-M_{A}$ (wt.%:wt.%), respectively.

The morphology and shape of the hydrogel nanoparticles poly$\left(M_{C}-co-M_{A}\right)$ after treatment with an electrolyte were examined by SEM analysis (Figure 3). In principle, the micrographs for both morphologies show evidence of aggregated irregular nanoparticles with rough surfaces (Figure 3a–c). It was observed as the concentration of thermo-sensitive groups (amide, –CONH_2_) increases; the nanoparticles tend to agglomerate with the addition of ${CaCl}_{2}$. This is because the ionic strength of the divalent$\;{CaCl}_{2}$ solution leads to a decrease in the osmotic pressure difference and the reduction in the driving swelling forces when the hydrogels interact with the calcium ions $\left({Ca}^{2+}\right)$, promoting the formation compact complexes. Additionally, due to systems that were maintained at low pH (<5) during the titration process, the polymeric chains tend to adopt a compact globular conformation [32,33,34]. Interestingly, wrinkled nano-sheets are observed in Figure 3d. Firstly, this is attributed to the interaction of the calcium ions with the pH-sensitive groups (carboxylic, –COOH), which are inside the particle. Subsequently, as a result of increasing the thermo-sensitive group (amide, –CONH_2_) content in the hydrogel, the morphology and structure of nanoparticles are transformed from a porous surface to a lamellar structure due to polymeric chains suffering a conformational change when the cations formed interact with the amide groups. That is, when the concentration of calcium ions is increased enough to screen the internal electrostatic attractions, the ionic bonds are broken. This means that the segments of the polymer are released and change from complex to free chains [35]. Finally, the particles are agglomerated, and, in some cases, the polymer chains extend enough to form irregular flakes. These interaction behaviors are also confirmed by the zeta potential (ζ) values discussed above.

### 2.4. Study of Rheological Properties at Different Temperatures of Poly$\left(M_{C}-co-M_{A}\right)$ Nanoparticles

**Figure 4:**
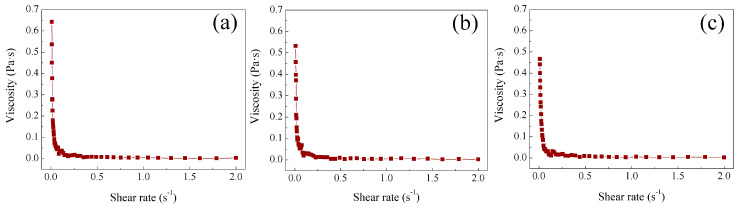
Viscosity$\;\left(\eta \right)\;$ as function of shear rate ($\dot{\gamma }$) of polymeric particles 50:50 of $\;M_{C}-M_{A}$ (wt.%:wt.%) for Serie 1 $\left(S_{1}\right)\;$at different temperatures: (**a**) 30 °C, (**b**) 50 °C and (**c**) 60 °C.

Rheological properties of the synthesized polymeric hydrogels of poly$\left(M_{C}-co-M_{A}\right)\;$were measured at several temperatures and compared for both particle morphologies, i.e., core–shell (Serie 1) and gradient (Serie 2). Figure 4 shows the viscosity $\;\left(\eta \right)\;$ at three specific temperatures (30 °C, 50 °C and 60 °C) for the polymer dispersion, with 50:50 of $M_{C}-M_{A}\;$ (wt.%:wt.%) for Serie 1$\left(S_{1}\right)$. It is essential to note the sample was chosen as an example because it represents the consistent behaviors over Serie 1. A dependence of viscosity$\;\left(\eta \right)\;$on low shear rates was observed where viscosity exhibited a significant decrement as the shear rate$\;\left(\dot{\gamma }\right)\;$increased within the range of 0.01 ≤ $\dot{\gamma }$/s^−1^ ≤ 0.5. This phenomenon is attributed to several factors, including structural changes in the polymeric chains of the hydrogel, such as the disintegration of agglomerates or alterations in particle shape during shearing. Additionally, the efficiency of intermolecular forces among polymeric chains diminished, resulting in reduced flow resistance. This rheological behavior aligns with the characteristics of a shear-thinning fluid. When the shear rate value rises to 0.5 s^−1^, the viscosity remains constant ($\eta_{\infty }$ = 2 mPa·s) and the flow resistance is reduced to a minimum value that cannot be decreased any further, corresponding to the friction between the uncoiled polymeric chains aligned in the driving force direction, describing now a Newtonian behavior. Latex can be described as a shear load-dependent fluid, exhibiting a non-Newtonian behavior at low shear rates and transitioning to a Newtonian behavior in the mid- to high-shear-rate range [36,37].

Evidently, a consistent shear load-dependent fluid behavior is observed at all three different temperatures, as depicted in Figure 4a–c. The primary temperature influence is seen in the initial viscosity value$\;{(\eta }_{0})\;$as the shear rate approaches zero. A decrease in viscosity is observed as the temperatures increases, as seen in the following:$\;\eta_{0}$ = 0.64 ± 0.01 Pa·s at 30 °C,$\;\eta_{0}$ = 0.532 ± 0.014 Pa·s at 50 °C and$\;\eta_{0}\;$= 0.466 ± 0.009 Pa·s at 60 °C. These results can be attributed to the impact of the low critical solution temperature$\;\left(LCST\right)\;$on the temperature-dependent average diameter$\;\left(D_{Z}\right)\;$of the hydrogel particles. Below ~32 °C (LCST of thermo-sensitive groups derivates) [38], the particle is swollen, and above this temperature the particle contracts by creating/breaking hydrogen bonds, a correlation that has been detailed in a previous work [22]. Hence, a larger particle size means increasing the polymer–polymer interaction, consequently leading to an increased resistance to the flow of the hydrogel [22].

**Figure 5:**
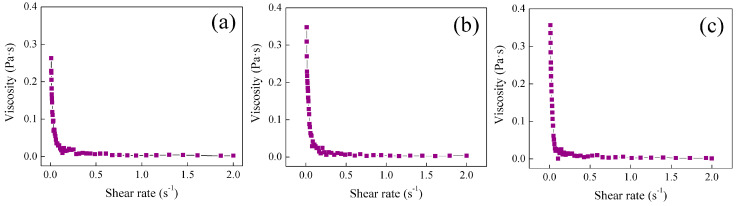
Viscosity $\left(\eta \right)$ as function of shear rate ($\dot{\gamma }$) of polymeric particles 50:50 of $M_{C}-M_{A}$ (wt.%:wt.%) for Serie 2 $\left(S_{2}\right)$at different temperatures: (**a**) 30 °C, (**b**) 50 °C and (**c**) 60 °C.

Viscosity analysis for the hydrogel with a 50:50 of $M_{C}-M_{A}$ (wt.%:wt.%) for Serie 2 $\left(S_{2}\right)$ is shown in Figure 5a–c as an example because all polymeric hydrogels have the same behavior.

The presence of a shear load-dependent fluid behavior is evident in the polymer hydrogel, emphasizing that the morphology of the particles does not apply a significant impact on this property. Nevertheless, a noticeable variation in viscosity is discernible as a function of temperature. In contrast to Serie 1 $\left(S_{1}\right)$, where viscosity decreased with a rising temperature, in Serie 2 $\left(S_{2}\right)$ the initial viscosity value ($\eta_{0}$) experiences an increase as the temperature elevates, as follows:$\;\eta_{0}$= 0.263 ± 0.051 Pa·s at 30 °C,$\;\eta_{0}$ = 0.348 ± 0.008 Pa·s at 50 °C and$\;\eta_{0}$ = 0.357 ± 0.101 Pa·s at 60 °C. This phenomenon is again attributed to the relationship between the average particle diameter $\;\left(D_{Z}\right)\;$ and the temperature. However, in this case, it is associated with the upper critical solution temperature$\;\left(UCST\right)\;$that corresponds to ~25 °C for the pH-sensitive (carboxylic, –COOH) and thermo-sensitive (amide, –CONH_2_) responsive groups inside of particles. Therefore, above this temperature, the particle is swollen by the complex dissociation of polymeric chains, which improves the motion of chains of the hydrogel [22].

The structural strength of the particles was evaluated by determining the viscoelastic properties as functions of temperature (30 °C, 50 °C and 60 °C). This viscoelastic behavior is characterized by the following two key moduli: the storage modulus (G′) and the loss modulus (G″). The storage modulus (G′) is often referred to as the elastic modulus and serves as a quantification of the energy stored by the sample during the shearing process. It reflects the capacity of the material for reversible deformation, essentially measuring its ability to regain its original form after experiencing stress. On the contrary, the loss modulus (G″), also known as the viscous modulus, quantifies the energy dissipated by the sample during the shearing process. Unlike the storage modulus, this energy is permanently lost to the material, signifying an irreversible deformation behavior. The loss modulus is a crucial indicator of the tendency of the material to undergo permanent deformation under applied stress [37]. 

**Figure 6:**
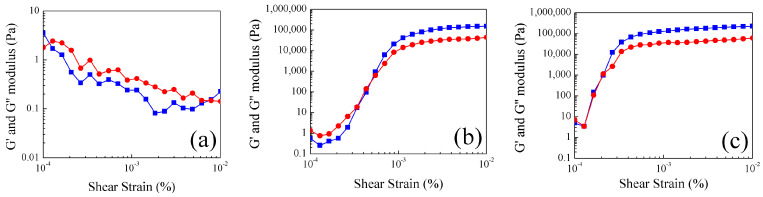
(■) Storage modulus (G′) and (●) loss modulus (G″) as a function of shear strain $\left(\gamma \right)$ of polymeric particles 50:50 of$\;M_{C}-M_{A}\;$(wt.%:wt.%) for Serie 1 $\left(S_{1}\right)$ at different temperatures: (**a**) 30 °C, (**b**) 50 °C and (**c**) 60 °C.

Storage (G′) and loss (G″) moduli as functions of shear strain (γ) at 30 °C, 50 °C and 60 °C for the hydrogel with a core–shell morphology are shown in Figure 6. At 30 °C, a distinct trend is observed where the loss modulus (G″) consistently surpasses the storage modulus (G′) across the entire shear strain range (Figure 6a). The G″ > G’ condition is indicative of liquid-like behavior. This is due to the ease with which stress can disrupt and break the weak hydrogen bonds within the core phase; these bonds maintain the particles in a swollen state at their lower temperatures.

In comparing 50 °C and 60 °C, the structural characteristics are similar between them (Figure 6b,c), respectively. Both hydrogels initiate with G″ > G′, and these moduli decrease in parallel at low shear strains. Subsequently, both moduli increase until they intersect at a specific point (G′ = G″), and from that point onwards, G′ predominates over G″, signifying an elastic and solid-like behavior. This condition persists, resulting in constant moduli values (G′ = 180 ± 30 kPa·s) which indicate a stable structure. Analyzing the crossing point, it is assumed that materials characterized at 60 °C turn into stable and solid-like structures with lower stress applied than those evaluated at 50 °C.

**Figure 7:**
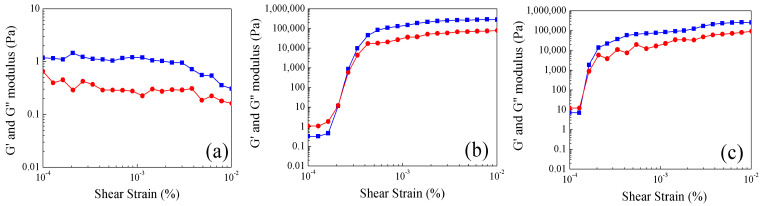
(■) Storage modulus (G′) and (●) loss modulus (G″) as function of shear strain$\left(\gamma \right)$ of polymeric particles 50:50 of$\;M_{C}-M_{A}\;$(wt.%:wt.%) for Serie 2 $\left(S_{2}\right)$ at different temperatures: (**a**) 30 °C, (**b**) 50 °C and (**c**) 60 °C.

Viscoelastic properties of nanoparticles poly$\left(M_{C}-co-M_{A}\right)$ with a concentration gradient morphology (Serie 2) were also investigated in the context of shear strain (γ) at identical temperature conditions for the analysis of core–shell structures (Serie 1). Firstly, a similar behavior was observed in Serie 2 as in Serie 1 (Figure 7). Nonetheless, the following two notable distinctions are worth highlighting: (i) At 30 °C, there is a marked solid-like behavior (G′ > G″) all over the strain range (Figure 7a) for the particles with a gradient morphology. This stability stands in contrast to the core–shell material behavior at the same temperature. (ii) When the emulsions are evaluated at 50 °C and 60 °C (Figure 7b,c), a similar trend is observed. Initially, there are plateaus or constant values at low shear strains (γ) followed by an increment of viscoelastic properties, leading to an intersection point (G′ = G″). This structural analysis of the polymeric hydrogels labels the particle characteristics as stable solids throughout the entire shear strain range.

**Figure 8:**
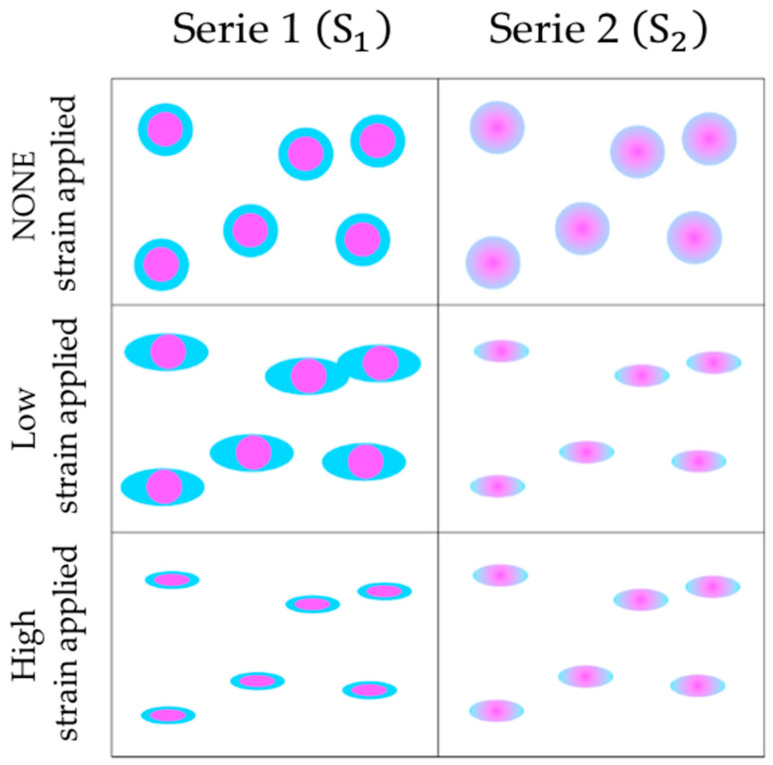
Effect on deformations for strain applied to different particle morphologies. Where: ∎ methyl methacrylate $\;\left(M_{M}\right),\;∎acrilyc\;acid\left(M_{C}\right),\;∎\;acrylamide\left(M_{A}\right)\;and$ 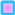 ${M_{C}-co-M}_{A}$.

On the contrary, with the core–shell structure behavior discussed above, there is an indication of an inverse shoulder at the same low shear strain (γ). Then, this feature can be attributed to the shell containing pH-sensitive (carboxylic, –COOH) and thermo-sensitive (amide, –CONH_2_) groups absorbing the initial deformation until the applied strain becomes sufficiently strong to reach the phase with more content of pH-sensitive groups. Subsequently, the solid particle transitions into a stable solid. Overall, this analytical contrast is aptly depicted in Figure 8. 

### 2.5. Analysis of Poly$\left(M_{C}-co-M_{A}\right)$ Nanoparticles by Nuclear Magnetic Resonance $\;\left(NMR\right)$

**Figure 9:**
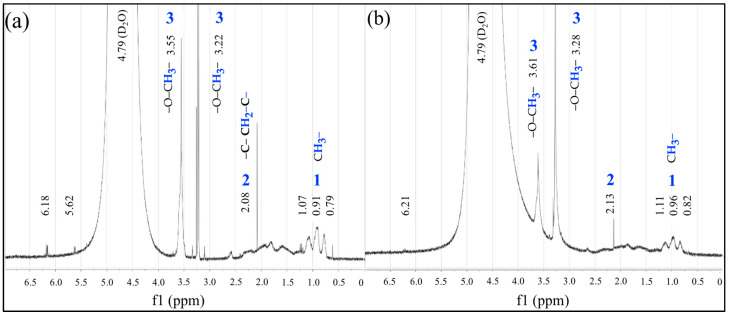
^1^H NMR spectra of polymeric particles 50:50 of $\;M_{C}-M_{A}$ (wt.%:wt.%) for (**a**) Serie 1 $\left(S_{1}\right)\;$and (**b**) Serie 2 $\left(S_{2}\right)$.

The ^1^H NMR spectra of polymeric hydrogels with a 50:50 ratio of$\;M_{C}-M_{A}\;$(wt.%:wt.%) for both morphologies are shown in Figure 9. At first, the spectra show three signals around 1 ppm attributed at methyl (C**H_3_**–) attached to carbon (C**H_3_**–C–) of the main chain bonded to the monomer methyl methacrylate$\;\left(M_{M}\right)$. In addition, two signals at 2.08 and 2.13 ppm are related to the methylene located in the primary chain (–C–C**H_2_**–C–), and then two intense signals at 3.22 ppm and 3.55 ppm are attributed to the methoxyl protons (–O–C**H_3_**–) of two polymeric chains with different molecular weights. On the other hand, the wide signal around 4.79 ppm belongs to the deuterium oxide$\;\left(D_{2}O\right)\;$and the last two weak signals around 6.21 and 6.18 ppm correspond to traces of non-polymerized reagents. Each of these signals belongs to the methyl methacrylate, and acrylic acid and acrylamide are indicated in both spectra at different intensities.

**Figure 10:**
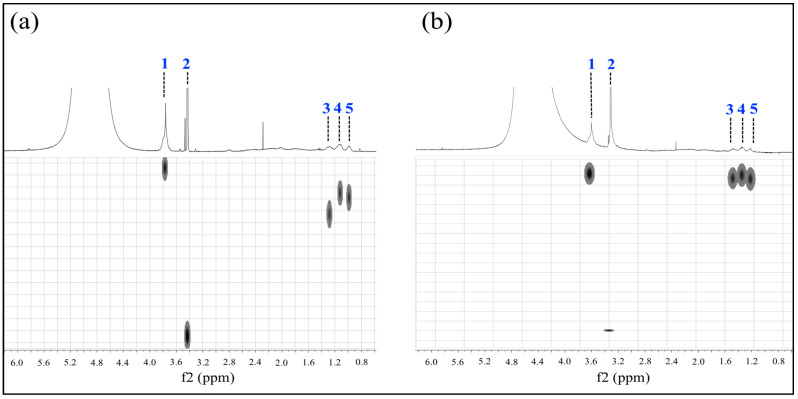
DOSY−NMR spectra of polymeric particles 50:50 of $M_{C}-M_{A}$ (wt.%:wt.%) for (**a**) Serie 1 $\left(S_{1}\right)\;$ and (**b**) Serie 2 $\left(S_{2}\right)$ at 25 °C.

DOSY experiments of polymeric particles poly$\left(M_{C}-co-M_{A}\right)$ for both structures were made at 25 °C, 30 °C and 35 °C. The results of this analysis at 25 °C are presented in Figure 10, with (a) Serie 1 $\;\left(S_{1}\right)\;$and (b) Serie 2 $\left(S_{2}\right),\;$because the polymeric hydrogels exhibit the same behavior throughout the temperature range. The obtained experimental data were normalized by a mathematical fitting in the Dynamic Program software (version 2.8.3), where five main signals were identified.

**Figure 11:**
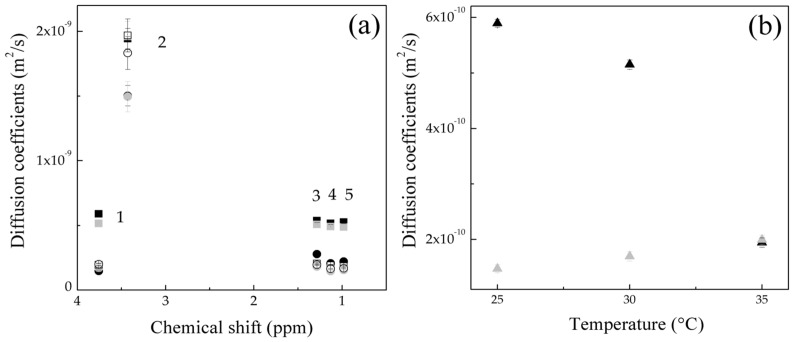
(**a**) Diffusion coefficients$\;\left(D\right)\;$ obtained by DOSY−NMR as function of chemical shift and temperature at 25 °C: (●) Serie 1 and (■) Serie 2, 30 °C: (●) Serie 1 and (■) Serie 2 and 35 °C: (○) Serie 1 and (☐) Serie 2 for all signals and (**b**) average diffusion coefficients as function as a function of temperature for signal 1: (▲) Serie 1 and (▲) Serie 2.

From DOSY data, the diffusion coefficient$\;\left(D\right)\;$as a function of chemical shift and temperature for Serie 1 and Serie 2 for several temperatures is obtained and shown in Figure 11a,b. Detailed data for each signal are shown in Table 1.

**Figure 12:**
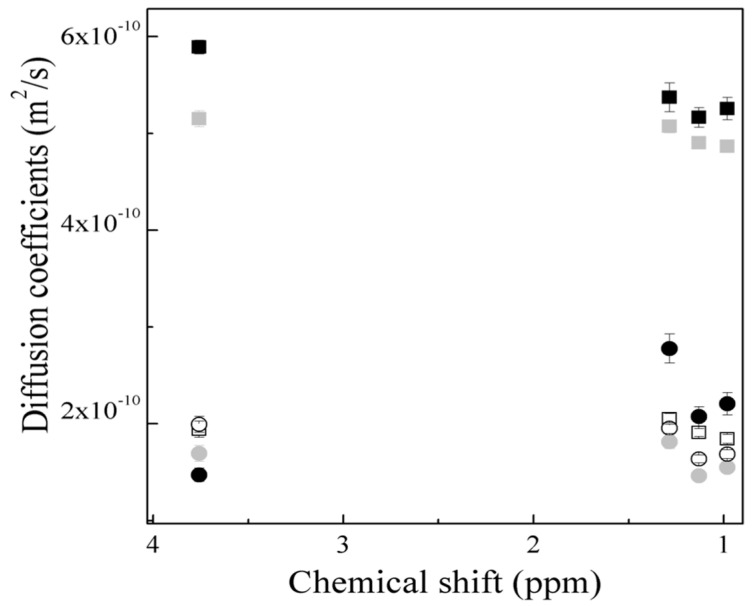
Diffusion coefficients$\;\left(D\right)\;$obtained by DOSY−NMR as function of chemical shift and temperature at 25 °C: (●) Serie 1 and (■) Serie 2, 30 °C: (●) Serie 1 and (■) Serie 2 and 35 °C: (○) Serie 1 and (☐) Serie 2.

In principle, higher values of the diffusion coefficients$\;\left(D\right)\;$corresponding to the signal 2 (≤ 2.0 × 10^−9^ $m^{2}\cdot s^{-1}$ ± 1.07 × 10^−10^) are observed for both morphologies. This is attributed to signal 2, which belongs to a polymeric chain with a low molecular weight and, therefore, showed higher diffusion coefficients than signals 1, 3, 4 and 5, which exhibited low diffusions caused by the high molecular weights of the polymer hydrogels (Figure 11a). For the core–shell structure, the obtained diffusion coefficients$\;\left(D\right)\;$data were analyzed separately in Figure 12. Signals 1, 3, 4 and 5 do not show significant differences in the diffusion phenomenon as a function of temperature.

Otherwise, signal 2 shows lower diffusion at 25 °C and 30 °C, while, at 35 °C, the diffusion values increase, indicating a decrement in the particle size (Figure 11a). This behavior is associated with the effect of shrinkage and/or dehydration of the hydrogels explained by the LCST of the thermo-sensitive groups (amide, −CONH_2_) on the surface of the particles [39], and it is also corroborated by the low resistance to flow observed in the decrease in the viscosity$\;\left(\eta \right)\;$and the liquid-like behavior of the polymeric material by increasing the temperature during the rheological analysis results shown in Figure 4 and Figure 6 for Serie 1$\left(S_{1}\right)$.

In analyzing the diffusion coefficients $\left(D\right)$ of the polymeric nanoparticles poly$\left(M_{C}-co-M_{A}\right)$ with a gradient concentration in Figure 12, it is important to note that the diffusion for signal 1, 3, 4 and 5 do not strongly depend on the temperature change. However, at 35 °C, there is a decrement in the diffusion coefficient values, suggesting an increment in the polymer chain size. The slight increment in particle size when the temperature rises is driven by the UCST (from a hydrophobic to−hydrophilic transition), leading to the dissolution of the particles, and this behavior is corroborated by the average hydrodynamic diameter $\left(D_{z}\right)\;$data obtained previously [22,40].

This phenomenon is also confirmed by the increase in viscosity$\;\left(\eta \right)\;$values and the solid-like behavior of the hydrogels when the temperature rises, as shown in Figure 5 and Figure 7 for Serie 2 $\left(S_{2}\right)$. This means that the polymer systems have a greater resistance to flow at temperatures above their UCST. On the other hand, for signal 2, the diffusion coefficient values increase with the increment of temperature (Figure 11a).

Parallelly, the diffusion coefficients of each signal were plotted as a function of temperature, where signal 1 is described as an example (Figure 11b). In Serie 1 $\left(S_{1}\right),$ it is observed that there is a tendency for diffusion coefficient $\left(D\right)$ values to increase as the temperature rises. Hence, when the critical minimum solution temperature is exceeded, the particles decrease in size, whereas the solvent is expelled from inside, therefore increasing the diffusion of the polymer in the medium. This means that as the kinetic energy is increased, the hydrogen bonds are broken and the thermoresponsive particle contracts, as described above [41,42]. Instead, Serie 2 $\left(S_{2}\right)$ showed a decrement in the particles diffusion when the critical maximum solution temperature was exceeded as a consequence of the increment of the kinetic energy and the generation of hydrogen bonds between the pH-sensitive (carboxylic, –COOH) and thermo-sensitive (amide, –CONH_2_) functional groups and the molecules of the medium, increasing the solubility of the particle [41,42]. The dependence between the change in the hydrodynamic diameter $\left(D_{H}\right)$, viscosity $\left(\eta \right)$ and diffusion coefficients $\left(D\right)$ with the variation of the temperature is associated with the results obtained previously [22] and the rheological data discussed above (Figure 4, Figure 5, Figure 6, Figure 7 and Figure 8). This means that, when the environmental parameters and morphology are modified, the hydrogels show conformational changes in their structure and their properties, such as viscosity $\left(\eta \right)$, storage (G′) and loss (G″) modulus.

Dynamic properties such as diffusion are measurements of the average displacement of molecules caused by the Brownian motion in the absence of external driving forces [43]. Specifically, diffusion coefficients of molecules or particles in a liquid phase are estimated based on the Stoke-Einstein Equation (1) as a function of the viscosity fluid and the mobility is related to the size of the particle through its hydrodynamic diameter [43,44]. Hence, the approach assumes that there are spherical particles diffusing in a liquid phase consisting of molecules small enough for the phase to be considered as continuum [44].

In this work, the diffusion coefficients $\left(D\right)$ were estimated from DLS data obtained in a previous work [22] by the following equation:

(1) $$D=\frac{kT}{3\pi \eta D_{H}}$$

where D is the particle diffusion coefficient, k is the Boltzmann constant (1.380649 × 10^−23^ J·K^−1^), T is temperature, η is the dynamic (shear) viscosity and$\;D_{H}$ is the hydrodynamic diameter of the particle [43,44,45]. The relationship between the measured diffusion coefficient $\left(D\right)$ values by NMR and DLS data at several temperatures are shown in Table 2. The observed diffusion coefficients$\;\left(D\right)\;$exhibit different orders of magnitude due to the distinct capabilities of the measurement techniques employed. DLS is suited for larger species with high molecular weights, whereas NMR is better suited for smaller structures with low molecular weights. Specifically, larger particles yield shorter proton transverse relaxation times, rendering them undetectable by NMR and resulting in higher diffusion coefficients [46]. Furthermore, different factors such as the concentration gradient, density, dispersion medium, viscosity$\left(\eta \right)$, particle size and temperature also contribute to differences in measurement values. For instance, typical diffusion coefficients for solids at ambient conditions are of the order of ${10^{-11}m}^{2}\cdot s^{-1}$, whereas those for liquids and gasses are three and seven orders higher [47,48].

On the other hand, the movements of the particles grow when the size of the hydrogel is decreased or the temperature is increased [48]. This means that the different values of diffusion are also attributed to the change in the conditions of analysis (dilution, proportions, solvent) and to the accuracy of the equipment of both characterization techniques (DLS and NMR). However, the calculated values showed a similar trend, although the order of magnitude varied.

According to the data presented in Table 2, Serie 1 shows the same increasing trend for the diffusion coefficient values obtained by NMR and DLS. This behavior is caused by the contraction of the particles when the temperature rises above ~32 °C $\left(LCST\right)$, resulting in an increase in the diffusion and a decrease in the viscosity values of the hydrogels. However, Series 2 does not exhibit significant changes in the diffusion results of the polymeric particles obtained by both characterization techniques despite increasing the temperature; as a result, the values remain almost constant at 25 °C and 30 °C. This corresponds to the change in analysis temperature not being sufficiently above the UCST (~25 °C) of the copolymer [11]. Therefore, there is no relevant conformational change in the particles except at 35 °C, where there is a slight decrease in the coefficient values that is more notable in the data obtained by DLS. This behavior is confirmed by the obtained results at constant values of the average particle diameter at the analysis temperature, as detailed in the latest research work [22]. In addition, according to the obtained results, the particle sizes of Serie 2 are bigger, which generated slower and lower diffusions. Hence, polymer solutions showed a decrease in their ability to flow according to the rheological properties studied above. It is relevant to mention that the diffusion coefficient data calculated by DLS are an average of the values obtained in triplicate during each analysis.

## 3. Conclusions

This study demonstrates that polymeric hydrogels$\;\left(M_{C}-co-M_{A}\right)\;$with dual sensitivity and distinct morphologies exhibit conformational changes in their structure in response to variations in pH and temperature. The average particle diameter$\;\left(D_{z}\right)\;$is significantly influenced by the critical solution temperature, resulting in swelling or shrinkage of the particles as a consequence of altered hydrophilic–hydrophobic interactions between the polymer chains and the solvent molecules. However, the addition of electrolyte salt does not affect particle diameter due to the absence of deprotonation of the carboxylic groups (–COOH) and electrostatic repulsions at a pKa of 4.5. Increasing the concentration of pH-responsive groups enhances stability of the hydrogels, while interaction with calcium ions $\left({Ca}^{2+}\right)$ decreases the colloidal stability induced by the growth of intermolecular interaction forces. The morphology of particles is determined by the ratio of pH- and thermo-sensitive groups, with spherical particles forming at higher carboxylic group concentrations and irregular porous morphologies forming with higher amide group contents. Although treatment with a calcium chloride $\left({CaCl}_{2}\right)$ solution leads to aggregated polymeric particles due to lost repulsive forces between polymer chains, rheological properties show a strong dependence on applied stress and temperature, modifying the flow capacity of the polymer materials by the increase or breaking of hydrophobic–hydrophilic interactions. ^1^H NMR and DOSY−NMR analyses confirm the presence of the protons of each monomer and a temperature-dependent diffusion coefficient$\;\left(D\right)$, indicating particle size changes due to swelling or contraction processes. This research highlights the potential of pH- and thermo-responsive polymeric hydrogels as ideal carriers for drug delivery in several medical treatments and paves the way for developing novel materials for biomedical and bioengineering applications.

## 4. Materials and Methods

The used chemicals in this research with some details and specifications are summarized in Table 3. All reagents were used as received without further purification.

### 4.1. Synthesis of Polymer Nanoparticles Poly$\left(M_{C}-co-M_{A}\right)$

**Figure 13:**
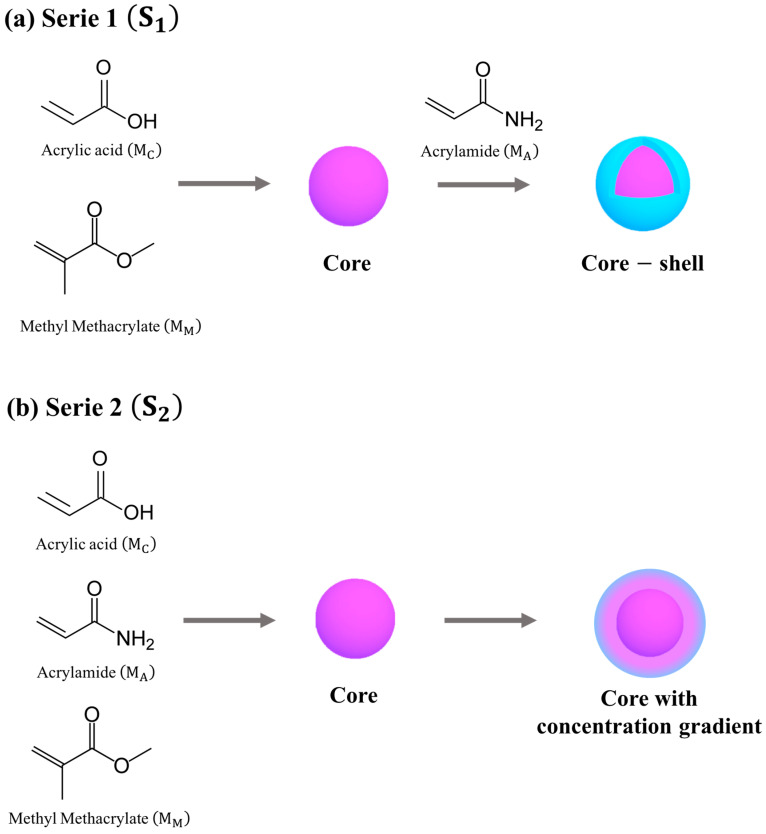
Schematic illustration of polymer particles morphology prepared by emulsion polymerization: (**a**) Serie 1 $\left(S_{1}\right)$: core–shell and (**b**) Serie 2 $\left(S_{2}\right)$: core with a concentration gradient. Where: ∎ methyl methacrylate$\;\left(M_{M}\right),\;∎acrilyc\;acid\left(M_{C}\right),\;∎\;acrylamide\left(M_{A}\right)\;and$ 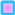 ${M_{C}–co–M}_{A}$.

Polymeric particles poly$\left(M_{C}-co-M_{A}\right)\;$were synthesized by an emulsion polymerization technique under the homogenous conditions given in Table 4. The pH-sensitive (acrylic acid,$\;M_{C}$) and thermo-sensitive (acrylamide, $M_{A}$) groups were changed over a wide concentration range, 100:0 wt.%–0:100 wt.%, at 10 wt.% intervals. According to the proposed synthesis, the polymeric hydrogels were prepared with total functional groups of 40 wt.%. The hydrogels synthesis was carried out to obtain two different morphologies (Figure 13). Serie 1$\;\left(S_{1}\right)$ was designed to obtain core–shell structured particles through a semicontinuous process. A high concentration of carboxylic (−COOH) groups were in the core of the particle, while the shell contained only a high proportion of amide (−CONH_2_) groups (Figure 13a). For the synthesis of Serie 2 $\left(S_{2}\right)$, a concentration gradient of$\;M_{C}-M_{A}$ has been generated in the particle by means of a power feed semicontinuous process (Figure 13b). Finally, the corresponding synthesis routes of both series were described in detail previously [22].

### 4.2. Latex Characterization

Polymer hydrogels were analyzed through use of several techniques as part of ongoing studies into their behavior, structural changes and interactions with the surrounding environment through pH- and thermo-sensitivity.

#### 4.2.1. Dynamic Light Scattering (DLS) and Zeta Potential (ζ)

The distribution particle diameter and the zeta potential (ζ) of hydrogels poly$\left(M_{C}-co-M_{A}\right)$ were measured at temperatures 25 ≤T/°C≤ 60 at 10 °C intervals.

The measurements were made using a Zetasizer Nano ZSP of Malvern Instruments (Malvern, UK). The polymer colloids were diluted at 10 ppm with deionized water from a stock solution. Before starting the measurement, 10 mL of diluted latex were placed in a tube for 10 min to equilibrate thermally, and then they were titrated with calcium chloride solution $\left({CaCl}_{2}\right)$ at 25 °C over the concentration range 0 ≤M/mM≤ 1.5 using a Multi–Purpose Titrator (MPT-2) attached from Malvern Instruments (Malvern, UK). For each titration, the change in average particle diameters$\;\left(D_{z}\right)$, zeta potential (ζ) and pH values were measured. After the analyses, the data were collected and analyzed to calculate the average particle diameters $\left(D_{z}\right)$ and the polydispersity index $\left(PDI\right)$. The assays were performed in triplicate. Measurements were made by adjusting and calibrating the equipment following the manufacturer protocol.

#### 4.2.2. Scanning Electron Microscopy $\left(SEM\right)$

Scanning electron microscopy$\;\left(SEM\right)\;$images of poly$\left(M_{C}-co-M_{A}\right)$ were obtained using JEOL JM-7800F Field Scanning Electron Microscope equipment, (Tokyo, Japan) operated at an accelerating voltage of ~5 kV, a working distance over the usual range of 4.0 ≤ $W_{D}/mm$ ≤ 10 and magnifications in the range from 10,000 to 50,000×. Initially, the samples were diluted with deionized water and dried onto the base of a cylindrical copper specimen holder for at least 1 h. After that, the polymeric hydrogels were covered with a thin layer of gold for at least 15 s to increase their electrical conductivity. Finally, images of samples both non-titrated and titrated with calcium chloride were obtained using the same operating conditions of the equipment.

#### 4.2.3. Rheology

Rheological properties analysis was conducted in a Modular Compact Rheometer model MCR 502 from Anton Paar (Graz, Austria). The viscosity $\left(\eta \right)$ for the polymeric particles poly$\left(M_{C}-co-M_{A}\right)$ was evaluated in rotation mode using a concentric cylinder measuring system (CC27). Samples of 18 mL for all hydrogels were characterized at temperatures 30 ≤ T/°C ≤ 60 and a shear rate ($\dot{\gamma }$) range of 0.01–100 s^−1^, though this range was restricted to 0.01–2 s^−1^ due to the constant viscosity $\left(\eta_{\infty }\right)$ behavior after this last value. Furthermore, a second set of experiments was performed in oscillatory mode to evaluate the structural strength as a function of temperature 30 ≤ T/°C ≤ 60. In this case, a cone and plate measuring system (CP25, 1°) was employed for 1 mL of latex using a gap of 0.054 mm, operating with a steady angular frequency (ω) of 1 rad·s^−1^ and a shear strain (γ) range of 0.0001–0.01%. All measurements were performed without diluting the sample and made in triplicate.

#### 4.2.4. Nuclear Magnetic Resonance $\left(NMR\right)$

NMR spectra were recorded on a Bruker 600 AVANCE III from Bruker BioSpin (Denver, CO, USA). Each of the emulsions were prepared by dissolving the sample to 600 μL in a 9:1 ratio of $H_{2}O$ (water) and $D_{2}O$ (Deuterium oxide), respectively. The spectra were internally referenced at 0 ppm to the singlet resonance of 1.0 mM TPS (triphenyl sulfonium). Finally, the measurements were made at 25 °C, 30 °C and 35 °C.

## Figures and Tables

**Table 1:** Diffusion coefficient $\left(D\right)$ values by DOSY−NMR for Serie 1 $\left(S_{1}\right)$ and Serie 2 $\left(S_{1}\right)$ at different temperatures $\left(T\right)$ for all signals $\left(s\right)$.

$\mathrm{Diffusion}\;\mathrm{Coefficients}\;\left(D,m^{2}/s\right)$		
(s)	$\mathrm{Serie}\;1\;\left(S_{1}\right)$	$\mathrm{Serie}\;2\;\left(S_{2}\right)$
T = 25 °C		T = 25 °C
1	1.47 × 10^−10^ ± 7.01 × 10^−12^	5.89 × 10^−10^ ± 7.01 × 10^−12^
2	1.50 × 10^−9^ ± 7.89 × 10^−11^	1.94 × 10^−9^ ± 7.89 × 10^−11^
3	2.78 × 10^−10^ ± 1.48 × 10^−11^	5.37 × 10^−10^ ± 1.48 × 10^−11^
4	2.07 × 10^−10^ ± 1.01 × 10^−11^	5.16 × 10^−10^ ± 1.01 × 10^−11^
5	2.21 × 10^−10^ ± 1.16 × 10^−11^	5.26 × 10^−10^ ± 1.16 × 10^−11^
	T = 30 °C	T = 30 °C
1	1.69 × 10^−10^ ± 8.41 × 10^−12^	5.15 × 10^−10^ ± 8.41 × 10^−12^
2	1.50 × 10^−9^ ± 1.16 × 10^−10^	1.96 × 10^−9^ ± 1.16 × 10^−10^
3	1.81 × 10^−10^ ± 6.69 × 10^−12^	5.07 × 10^−10^ ± 6.69 × 10^−12^
4	1.46 × 10^−10^ ± 4.12 × 10^−12^	4.90 × 10^−10^ ± 4.12 × 10^−12^
5	1.55 × 10^−10^ ± 5.47 × 10^−12^	4.86 × 10^−10^ ± 5.47 × 10^−12^
	T = 35 °C	T = 35 °C
1	1.99 × 10^−10^ ± 8.26 × 10^−12^	1.94 × 10^−10^ ± 8.26 × 10^−12^
2	1.83 × 10^−9^ ± 1.27 × 10^−10^	1.97 × 10^−9^ ± 1.27 × 10^−10^
3	1.96 × 10^−10^ ± 5.58 × 10^−12^	2.05 × 10^−10^ ± 5.58 × 10^−12^
4	1.64 × 10^−10^ ± 4.01 × 10^−12^	1.91 × 10^−10^ ± 4.01 × 10^−12^
5	1.68 × 10^−10^ ± 4.55 × 10^−12^	1.84 × 10^−10^ ± 4.55 × 10^−12^

**Table 2:** Diffusion coefficient $\left(D\right)$ values calculated by NMR and DLS for Serie 1 $\left(S_{1}\right)$ and Serie 2 $\left(S_{1}\right)$ at different temperatures $\left(T\right)$ for signal 2.

$\mathrm{Diffusion}\;\mathrm{Coefficients}\;\left(D,m^{2}/s\right)$		
T/°C	$\mathrm{Serie}\;1\left(S_{1}\right)$	$\mathrm{Serie}\;2\left(S_{2}\right)$
25	1.50 × 10^−9 a^	1.94 × 10^−9 a^
	5.82 × 10^−15 b^	5.86 × 10^−15 b^
30	1.50 × 10^−9 a^	1.96 × 10^−9 a^
	6.90 × 10^−15 b^	6.07 × 10^−15 b^
35	1.83 × 10^−9 a^	1.97 × 10^−9 a^
	8.40 × 10^−15 b^	5.78 × 10^−15 b^

Determined by ^a^  NMR and ^b^  DLS.

**Table 3:** Information on chemical materials.

Component	Source and Country	Mass Fraction Purity
pH-sensitive group: acrylic acid $\left(M_{C}\right)$	Sigma–Aldrich (Burlington, MA, USA)	≥98 ^a^
Thermo-sensitive group: acrylamide $\left(M_{A}\right)$	Sigma–Aldrich, (Shanghai, China)	≥98 ^a^
Monomer: methyl methacrylate $\left(M_{M}\right)$	Poliformas Plásticas (Mexico City, Mexico)	≥90 ^a^
Initiator: sodium persulfate $\left(I_{SP}\right)$	Sigma–Aldrich, (Burlington, MA, USA)	≥98 ^a^
Electrolyte: calcium chloride $\left({CaCl}_{2}\right)$	Sigma–Aldrich, (Tokyo, Japan)	≥93 ^a^
Deuterium oxide	Sigma–Aldrich, (Burlington, MA, USA)	≥99.99 ^a^
Surfactant: octylphenol ethoxylate	Solvay, (New York City, NY, USA)	- ^a^
Destilled water	Mizu Técnica (Naucalpan de Juárez, Mexico)	- ^b^

^a^ Reagent grade and ^b^ industrial grade.

**Table 4:** Operation conditions during synthesis of Serie 1 $\left(S_{1}\right)$ and Serie 2 $\left(S_{2}\right).$

Parameter	Values
Atmosphere: Nitrogen $\left(N_{2}/Psi\right)$ ^b^	~40
Temperature $\left(T/{}^{\circ}C\right)$	~75
Mechanical stirring (rpm)	~250
Flow rate $\left(V_{1};V_{2}/{g\cdot min}^{-1}\right)$	0.7

^b^ Industrial grade.

## Data Availability

The data presented in this study are openly available in the article.
